# Asthma Risk Prevalence and Associated Factors in Stunted Children: A Study Using Asthma Predictive Index

**DOI:** 10.3390/medicina61010140

**Published:** 2025-01-16

**Authors:** Gartika Sapartini, Gary W. K. Wong, Agnes Rengga Indrati, Cissy B. Kartasasmita, Budi Setiabudiawan

**Affiliations:** 1 Division of Allergy Immunology, Department of Child Health, Doctoral Study Program, Faculty of Medicine, Universitas Padjadjaran, Bandung 45363, West Java, Indonesia; budi.setiabudiawan@unpad.ac.id; 2 Department of Paediatrics, Faculty of Medicine, The Chinese University of Hong Kong, Hong Kong, China; wingkinwong@cuhk.edu.hk; 3 Department of Clinical Pathology, Faculty of Medicine, Universitas Padjadjaran/Dr. Hasan Sadikin General Hospital, Bandung 45363, West Java, Indonesia; agnes.indrati@unpad.ac.id; 4 Division of Respirology, Department of Child Health, Faculty of Medicine, Universitas Padjadjaran/Dr. Hasan Sadikin General Hospital, Bandung 45363, West Java, Indonesia; cbkarta@gmail.com; 5 Faculty of Medicine, President University, Bekasi 17550, West Java, Indonesia

**Keywords:** API score, asthma, stunted children, malnutrition, stunting

## Abstract

*Background and Objectives*: The prevalence of stunted children under 5 years in Indonesia is relatively high. Stunting is a significant risk factor for wheezing disorders. The asthma predictive index (API) identifies children with a recurrent wheezing disorder at risk of developing asthma during the first 3 years. However, the risk of developing asthma and its associated factors among stunted children has not been studied. This study aims to determine the asthma risk prevalence in stunted children via the API score and associated factors. *Materials and Methods*: This cross-sectional study was conducted at the Bandung District Health Center from October 2021 to January 2022. This study included stunted children aged 24–59 months living in Bandung District whose parents could answer the API and asthma risk factor questionnaires. *Results*: A total of 422 participants with an average age of 43.1 ± 9.7 months were included. Among the stunted children, 4.7% (20/422) met the positive API criteria, and 50.0% were malnourished (stunted–underweight). The participants with positive API results had a parental medical diagnosis of asthma (45%), eczema (10.0%), allergic rhinitis (20.0%), and wheezing apart from colds (40.0%) (*p* < 0.05). Significant risk factors for developing asthma in the participants with a positive API were dog ownership in the past 12 months and parents and siblings with allergic diseases. *Conclusions*: The asthma risk prevalence in stunted children was 4.7%. The associated risk factors included a history of allergic diseases in parents and siblings, as well as dog ownership; however, further investigation is needed.

## 1. Introduction

Asthma is a complex phenotype resulting from the interaction of diverse endotypes associated with diverse conditions, and it should ideally be submitted for differential diagnosis for personalized treatment [1,2].

Stunting results from chronic malnutrition and remains a global problem [3]. The worldwide incidence of stunted children is relatively high, especially in low- and middle-income countries. In Indonesia, the latest data from Basic Health Research (RISKESDAS) 2018 revealed that the prevalence of stunting among children under five years is 30.8% [4]. Hawlader et al. reported that among rural Bangladesh children, stunting was a significant risk factor for wheezing disorders [3]. Diet or nutritional status changes have been hypothesized to directly influence the immune system to induce an allergic reaction and asthma [5].

Stunted children have low lean body mass [6]. Lean body mass is significantly associated with lung function in underweight children [7]. Stunted–underweight children have lower lean body mass, impaired lung growth, and considerably lower lung function, which is associated with a greater incidence of asthma symptoms [8].

Malnutrition can reduce adipocyte mass, lowering circulating leptin levels [9]. This leptin deficiency may cause the immune response to shift toward Th2 cytokines, such as IL-4, and reduce Th1 cytokine levels. A study involving malnourished children revealed an imbalance in the type 1/type 2 immune response, with a significant decrease in serum leptin levels leading to an increase in IL-4 levels [10]. Although further investigation is necessary, IL-4 levels may increase the probability of asthma development [11].

Several main factors are associated with asthma development in children, including genetic predispositions, immunizations, diet, and exposure to allergens, pollutants, endotoxins, parasites, and viruses. Children under five years experience one episode of asthmatic symptoms, including wheezing, coughing, or dyspnea [12]. The incidence of current wheezing (wheezing symptoms in the past 12 months) in stunted children aged 4–5 years is 24.9%. Stunting is significantly associated with wheezing disorders (odds ratio [OR] = 1.74; 95% confidence interval [CI] = 1.19–2.56) [3], although the prevalence of asthma in stunted children has not been studied to date.

Asthma is a common chronic disease in children, with most cases beginning before five years of age [13]. However, asthma is still among the most challenging diseases for pediatricians to diagnose because the clinical symptoms of asthma in this age group are variable and nonspecific. There is no single “gold-standard” test that can be used to diagnose asthma accurately [14]. Therefore, asthma diagnosis in children aged <5 years is based on subjective clinical features and findings from physical examinations [14,15].

In 2000, the asthma predictive index (API) was developed to identify children with recurrent wheezing identified in the first 3 years who were at significant risk for developing asthma later in life [14,15]. The API is simple, inexpensive, noninvasive, and well validated [16]. It has been used in various countries to screen high-risk groups for asthma development [17]. Given that stunting is a risk factor for wheezing disorders and the need to screen stunted children who are at risk of developing asthma in the future, this study aimed to determine the prevalence of stunted children who are at risk for developing asthma later in life via the API and to evaluate its associated risk factors.

## 2. Materials and Methods

### 2.1. Study Design and Participant Selection

This cross-sectional study was conducted at the Bandung District Health Center from October 2021 to January 2022.

### 2.2. Data Collection

Altogether, 422 stunted children aged 24–59 months whose parents could answer the questionnaire and provide informed consent were randomly selected via multistage random sampling from 15 out of 62 public healthcare centers in Bandung District and included in the study.

This study used primary data obtained via anthropometric examinations (patient weight, height, and nutritional status), API (Table 1), asthma risk factor questionnaires, and examinations of eosinophils from peripheral blood. Those who met the inclusion criteria and those who did not meet the exclusion criteria were selected. The inclusion criteria were as follows: (1) stunted–underweight (HFA <−2 SD with WFA <−2 SD) and (2) stunted–wellnourished (HFA <−2 SD with WFA between −2 SD and 2 SD) according to the WHO child growth standard/WHOCGS curve.

**Figure 1:**
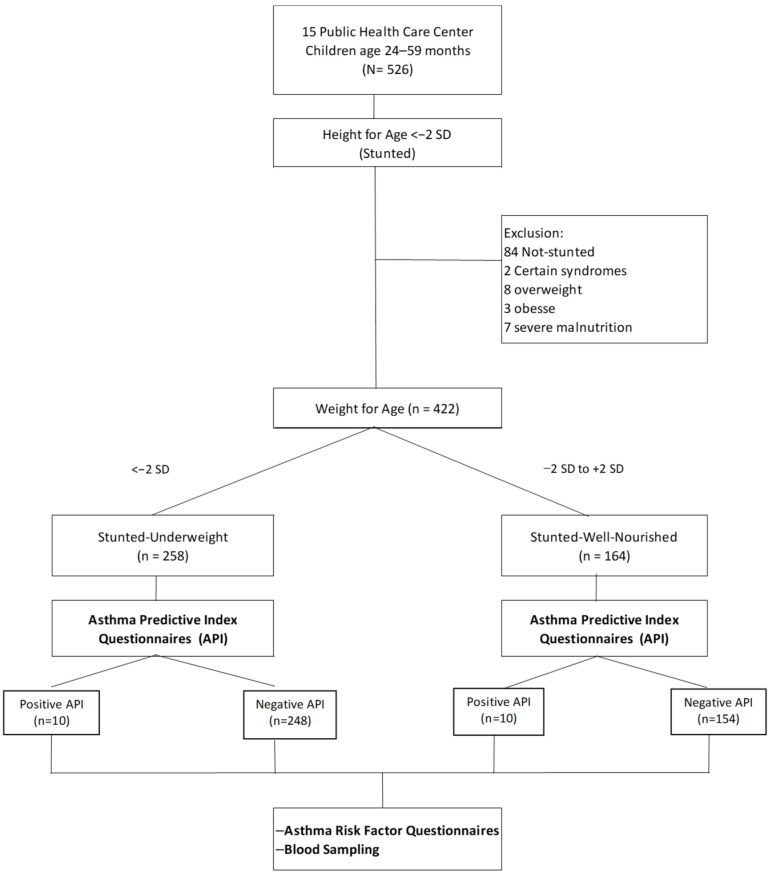
Flow chart of the selection of the study participants.

Figure 1 presents the flow diagram for analyzing asthma risk prevalence in stunted children aged 24–59 months from 15 public health centers.

#### 2.2.1. Initial Sample

A total of 526 children were screened for the study.

These children were assessed for their height-for-age to determine stunting (defined as height-for-age <−2 standard deviations (SD)).

#### 2.2.2. Exclusion Criteria

Children who did not meet the stunting criteria (*n* = 84), had certain syndromes (*n* = 2), were overweight (*n* = 8), obese (*n* = 3), or had severe malnutrition (*n* = 7) were excluded.

After exclusions, 422 children with stunting remained.

#### 2.2.3. Weight-for-Age Categorization

The remaining children were further classified based on their weight-for-age:

Stunted–underweight (weight-for-age <−2 SD): 258 children.

Stunted–wellnourished (weight-for-age between −2 SD and +2 SD): 164 children.

#### 2.2.4. Asthma Risk Assessment

Both groups underwent asthma risk assessment using the asthma predictive index (API) questionnaires.

Among the stunted–underweight group:

Positive API results: 10 children.

Negative API results: 248 children.

Among the stunted–wellnourished group:

Positive API results: 10 children.

Negative API results: 154 children.

#### 2.2.5. Further Analysis

All the study participants completed additional assessments, which included asthma risk factor questionnaires and blood sampling.

This diagram depicts the systematic screening and classification process, ensuring that the study meets its inclusion criteria and evaluates the prevalence of asthma risk in stunted children.

Sample size determination was adjusted according to the research objective, which was to determine the asthma risk prevalence in stunted children, and the sample size formula for a cross-sectional study (estimating population proportion) was used. In this study, a minimum sample of 287 stunted children was required.

During the study, the participant’s weight and height were measured, and their height was compared with the WHOCGS curve to determine whether they were stunted or not stunted by their age. In addition, the participants’ parents were interviewed via asthma predictive index (API) questionnaires and any associated risk factors. To minimize recall bias, the researcher guided questionnaire completion through in-person interviews conducted in quiet and comfortable settings. The presence of the primary caregiver was encouraged to assist with recall within the specified timeframe.

The participants were divided into four groups: stunted–underweight with positive API, stunted–underweight with negative API, stunted–wellnourished with positive API, and stunted–wellnourished with negative API. The participants who met the following exclusion criteria were not included in the study: (1) had a normal height (HFA >−2 SD); (2) had specific syndromes; (3) were overweight; (4) were obese; and (5) were severely malnourished (weight for age <−3 years).

Peripheral venous blood samples were collected from each group. They were sent to the Clinical Pathology Laboratory of Dr. Hasan Sadikin Hospital Bandung. The pooled blood samples were used to measure the level of eosinophilia. Figure 1 illustrates the selection process for the study participants.

### 2.3. Ethical Considerations

We obtained approval from the Ethics Committee of Padjadjaran University before initiating the study (number 572/UN6.KEP/EC/2021) and the participants’ parents signed an informed consent form before participating.

### 2.4. Anthropometric Measurements

We used a calibrated digital scale to measure the weight of children aged ≥2 years who could stand independently. The scale was placed on a flat and hard surface. We used a stadiometer with a fixed vertical backboard and an adjustable headpiece to measure height. For children aged ≥2 years who could stand independently without assistance, we assessed their maximum vertical size: their height. Stunting was identified when the height was <−2 SD from the median WHOCGS curve for individuals of the same age and sex [18].

### 2.5. API Questionnaires

The asthma predictive index (API) has been used to screen high-risk groups for asthma development. The API was developed from follow-up data from the Tucson population-based study to predict whether preschool children with wheezing develop asthma by the age of 6 years (Table 1) [14]. Positive API is ruled out by more than three episodes of wheezing plus one or more major criteria [eczema; parental asthma] or two or more minor criteria [allergic rhinitis; eosinophilia; wheezing apart from colds]. A negative API was ruled out in addition to those criteria.

### 2.6. Blood Eosinophil Measurement

Eosinophils are a type of white blood cell, and their percentage of the total white blood cell count is measured through a complete blood count (CBC) examination at the Clinical Pathology Laboratory of Dr. Hasan Sadikin Hospital in Bandung. Two milliliters of venous blood with EDTA anticoagulant was added to the XN-1000^TM^ automated hematology analyzer by Sysmex Corporation, Kobe, Japan. The results are compared to a reference range, indicating that the normal range of eosinophils in the blood is 0–4%. A higher-than-normal eosinophil count indicates eosinophilia.

### 2.7. Statistical Analysis

After collecting all the data, we analyzed the API result, exposure to allergens, family history of allergies, history of childbirth, and breastfeeding. The data were analyzed via Pearson’s chi-square or Fisher’s exact test. A *p*-value of less than 0.05 was considered significant for all the tests.

## 3. Results

### 3.1. Participant Characteristics

Table 2 shows that 422 participants, with a mean age of 43.1 ± 9.7 months, met the inclusion criteria for stunting. Among the participants, 224 (53.1%) were boys. Regarding height and weight, most of the study participants were stunted–underweight (61.1%). Table 3 shows that 4.7% (20/422 participants) of the stunted children had positive API, with 50% (10 participants) having stringent positive API and 50% (10 participants) having loose positive API. The same prevalence was observed between the stunted–underweight and stunted–wellnourished patients (10/20 participants). Based on the API criteria, all the criteria among the positive API participants were statistically significant (Table 4). Table 5 shows that the significantly associated risk factors for developing asthma in positive API participants were dog ownership at 0–12 months of age and in the past 12 months, parents with allergic diseases, and siblings with allergic diseases.

### 3.2. Risk Factors for Developing Asthma in Stunted Children

Positive API participants were more likely to have allergen exposures (cat and dog ownership) than negative API participants were, with dog ownership at 0–12 months of age and in the past 12 months being significant risk factors. The positive API group had a more frequent history of allergic disease in parents and siblings than did the negative API group (*p* ≤ 0.05). The percentages of participants born by cesarean delivery in the positive and negative API groups were 5% and 5.4%, respectively. The number of preterm deliveries among the participants with a positive API was higher than that of the participants with a negative API (10% vs. 7.7%). Most participants were born in both the positive and negative API groups at term. Most positive and negative API participants had a history of breastfeeding (85% and 84.3%, respectively).

## 4. Discussion

The API is a crucial tool for assessing the risk of asthma development in children under 5 years of age [16]. In the present study, we used the API to investigate stunted children aged 24–59 months who were at risk for developing asthma. Among the 422 participants, 20 stunted children (4.7%) had positive API (at risk for asthma). In our study, the number of stunted children who wheezed was lower than that reported in previous studies [3], as our research participants were from rural areas. This might be because exposure to agricultural farming confers the same protection against the subsequent development of asthma as livestock farming does [19].

Wheezing in young children can result from various conditions besides asthma, including viral infections, anatomical abnormalities, or transient wheezing due to airway hyperreactivity [3,19]. In this study, we specifically focus on wheezing associated with asthma rather than wheezing caused by other factors. Wheezing related to asthma is characterized by several key features: it is typically triggered by asthma stimuli such as allergens, exercise, cold air, or emotional stress; it improves with the use of bronchodilators; it has an episodic nature, meaning there are periods without wheezing in between episodes; and it responds positively to asthma control measures [19].

Among preschool children in Colombia, Rodriguez-Martinez et al. reported that the API has high specificity (79.2%). More extensive studies conducted in the Netherlands and England reported 92% and 93% specificities for the API [14,16,19]. The API has consistently high specificity. Thus, a positive API strongly predicts asthma in children with a history of wheezing [17].

Previous studies have reported that stunting is a significant risk factor for wheezing disorders in rural Bangladesh [3]. Stunting is considered a result of chronic malnutrition. Malnourished children had lower lung function and body fat, as well as a greater incidence of asthma symptoms. Normal lung growth may be impaired in malnourished children, which increases the chance of asthma symptom development [3]. In this study, 10 out of 20 positive API participants were malnourished (stunted–underweight).

Ten percent of the participants with positive API results had a history of atopic dermatitis diagnosed at 2–3 years of age. The Taiwan Birth Cohort Study revealed that among infants diagnosed with atopic dermatitis before 3 years of age, approximately 21.6% had asthma by 8 years [3,20].

Among the participants with positive API, 20% were diagnosed with allergic rhinitis at 2–3 years of age. An American study reported that children with an onset of allergic rhinitis in the first year of life had more respiratory symptoms at 6 years of age and were more likely to be diagnosed with asthma [21]. A history of allergic rhinitis was significantly associated with the occurrence of asthma (OR = 3.82; 95% CI: 2.92–4.99) [21].

The role of eosinophils in asthma pathogenesis has been extensively studied. In allergic eosinophilic asthma, eosinophils play a vital role as effector and antigen-presenting cells in allergic inflammatory reactions. In contrast, the airways’ type 2 innate lymphoid cells contribute significantly to eosinophil activation in nonallergic eosinophilic asthma. Although eosinophilia is more common in allergic asthma patients, it may also increase in some cases of nonallergic asthma [22]. Following previous research, our study revealed that individuals with a positive API had a significantly greater proportion of eosinophilia (15%) than those with a negative API (1.2%).

Taniguchi et al. reported that dog and cat ownership from infancy did not increase the risk of developing wheezing disorders and asthma compared with Japanese children who had never owned them [23]. In contrast to our findings, API-positive participants who had dogs in the past 12 months had a fairly high risk of asthma development [24]. However, some exposures associated with dogs might significantly augment the effect of air pollution [24,25]. Another study reported that sensitization to indoor inhalant allergens (i.e., dogs) was associated with more severe asthma symptoms in adults [26].

One of the factors that increases the risk of developing asthma is having a parent with a history of asthma. Children with a parent with asthma are almost twice as likely to develop asthma than those without a parent with asthma [27]. Forty-five percent of the participants with positive API in this study had parents with a history of allergic disease, with half of them having a history of asthma. A cohort study conducted in Taiwan reported that a parental history of asthma was a strong predisposing factor for asthma in children [21,28].

The Childhood Origins of Asthma study reported that participants who had siblings with asthma were more likely to have asthma at the ages of 6, 8, 11, and 13 years [13]. Another US study reported that 36–40% of the children diagnosed with asthma between 6 and 13 years of age had siblings with asthma [13,21]. In our study, 15% of the positive API participants had siblings with a history of allergic diseases. This percentage was lower than that reported in a previous study but greater than that of the negative API participants.

The number of preterm deliveries among the positive API participants was greater than that among the negative API participants (10% vs. 7.7%). This finding is consistent with that of Been et al., who reported that preterm birth was associated with a 1.46-fold increased risk of developing asthma or wheezing disorders. They also reported an equally strong association between preterm birth and wheezing disorders in children aged ≥5 years [13,21,28,29].

A previous cohort study reported that breastfeeding is associated with a lower risk of asthma in children aged <7 years [30,31]. Exclusive breastfeeding for at least 6 months prevents asthma or delays its onset [31]. However, the number of positive API participants who received breast milk was almost the same as that of negative API participants.

In this study, 4.7% of the stunted children were API positive, indicating that asthma risk will occur. When the API result is positive, the child is considered to be at high risk of developing asthma in the future [21,30,31]. Although it does not provide a definitive diagnosis of asthma, it is an early warning sign that the child may be more susceptible to developing asthma later in life. Regular follow-up with a healthcare professional is crucial to monitor a child’s respiratory health, reassess symptoms, and make appropriate treatment decisions based on their condition [13,21]. Healthcare providers may also recommend additional preventive measures and interventions to minimize the risk of asthma development [21,27]. The most important thing is to improve their nutritional status, followed by lifestyle modifications, environmental control measures, and, in some cases, the initiation of asthma medications or preventive treatments [21,27,30,31].

The major limitation of our study was its cross-sectional study design, which made it impossible to confirm whether the children developed asthma later in life. Moreover, there were several limitations in this study. The number of positive API participants was small. Besides that, the study sample comes from specific health centers only in Bandung District, raising concerns about its representativeness for broader populations. Further investigation with larger samples which includes participants from other geographic areas is needed to confirm and extend these findings. There may still be unmeasured or unexplained confounding factors in our study such as socioeconomic status, environmental factors (indoor air pollution and allergen exposure), and genetic predisposition.

## 5. Conclusions

Based on the API instruments, we determined that the prevalence of asthma risk in stunted children aged 24–59 months (positive API participants) was 4.7%. Approximately 50% of the positive API participants were malnourished (stunted–underweight). The risk factors associated with positive API include a history of allergic diseases in parents and siblings, as well as dog ownership.

## Figures and Tables

**Table 1:** Asthma predictive index ^†^ criteria.

Major Criteria	Minor Criteria
Parental MD asthma ^‡^	MD allergic rhinitis ^§^
MD eczema ^¶^	Wheezing apart from colds
	Eosinophilia (≥4%)

Abbreviation: MD, medical diagnosis. ^†^ loose index for asthma prediction: early wheezer and at least one of the two major or two of the three minor criteria. Stringent index for asthma prediction: early frequent wheezer and at least one of the two major or two of the three minor criteria. ^‡^ history of a physician diagnosis of asthma. ^§^ physician diagnosis of allergic rhinitis at 2–3 years of age. ^¶^ physician diagnosis of atopic dermatitis at 2–3 years of age.

**Table 2:** Participant characteristics.

Characteristic	Total (*n* = 422)	%
Sex		
Male	224	53.1
Female	198	46.9
Age, months		
24–35	114	27.0
36–47	144	34.1
48–59	164	38.9
Nutritional status		
Stunted–underweight	258	61.1
Stunted–wellnourished	164	38.9

**Table 3:** Asthma predictive index in stunted children.

Nutritional Status	Stunted– Underweight	Stunted–Well nourished	Total	*p*-Value *
Positive API	10 (50.0%)	10 (50.0%)	20 (4.7%)	0.295
Negative API	248 (61.7%)	154 (38.3%)	402 (95.3%)	

* based on the chi-square test.

**Table 4:** Comparison of asthma predictive index criteria between positive and negative API groups.

API Criteria	Positive API (*n* = 20)	Negative API (*n* = 402)	*p*-Value *
Parental MD asthma	9 (45.0%)	11 (2.7%)	<0.001
MD eczema	2 (10.0%)	5 (1.2%)	0.039
MD allergic rhinitis	4 (20.0%)	3 (0.7%)	<0.001
Wheezing apart from colds	8 (40.0%)	10 (2.5%)	<0.001
Eosinophilia	3 (15.0%)	5 (1.2%)	0.004

Abbreviations: MD, medical diagnosis. * chi-square test or Fisher’s exact test.

**Table 5:** Risk factors for developing asthma in stunted children.

Risk Factor	Positive API *n* (%)	Negative API *n* (%)	*p*-Value *
Allergen exposure (cat ownership)			
When the participant was 0–12 months old	2 (10.0)	16 (4)	0.207
In the past 12 months	2 (10.0)	11 (2.7)	0.122
Allergen exposure (dog ownership)			
When the participant was 0–12 months old	1 (5.0)	1 (0.3)	0.003
In the past 12 months	3(15.0)	1 (0.3)	<0.001
History of allergic diseases ** in parents	9 (45.0)	11 (2.7)	<0.001
History of allergic diseases ** in siblings	3 (15.0)	15 (3.7)	0.047
Cesarean delivery	1 (5.0)	22 (5.4)	1.000
Preterm delivery	2 (10.0)	31 (7.7)	0.663
Breastfeeding	17 (85.0)	339 (84.3)	1.000

* based on Fisher’s exact test. ** asthma, allergic rhinitis, atopic dermatitis, and food allergy.

## Data Availability

The data underlying this study’s findings are accessible from the corresponding author upon reasonable request.
